# Antenatal care and perinatal outcomes in Kwale district, Kenya

**DOI:** 10.1186/1471-2393-8-2

**Published:** 2008-01-10

**Authors:** Celia A Brown, Salim B Sohani, Khalid Khan, Richard Lilford, Walter Mukhwana

**Affiliations:** 1 Department of Public Health and Epidemiology, The University of Birmingham, UK; 2 Aga Khan Health Service, Kenya; 3 Birmingham Women's Hospital, UK

## Abstract

**Background:**

The importance of antenatal care (ANC) for improving perinatal outcomes is well established. However access to ANC in Kenya has hardly changed in the past 20 years. This study aims to identify the determinants of attending ANC and the association between attendance and behavioural and perinatal outcomes (live births and healthy birthweight) for women in the Kwale region of Kenya.

**Method:**

A Cohort survey of 1,562 perinatal outcomes (response rate 100%) during 2004–05 in the catchment areas for five Ministry of Health dispensaries in two divisions of the Kwale region. The associations between background and behavioural decisions on ANC attendance and perinatal outcomes were explored using univariate analysis and multivariate logistic regression models with backwards-stepwise elimination. The outputs from these analyses were reported as odds ratios (OR) with 95% confidence intervals (CI).

**Results:**

Only 32% (506/1,562) of women reported having any ANC. Women with secondary education or above (adjusted OR 1.83; 95% CI 1.06–3.15) were more likely to attend for ANC, while those living further than 5 km from a dispensary were less likely to attend (OR 0.29; 95% CI 0.22–0.39). Paradoxically, however, the number of ANC visits increased with distance from the dispensary (OR 1.46; 95% CI 1.33–1.60). Women attending ANC at least twice were more likely to have a live birth (vs. stillbirth) in both multivariate models. Women attending for two ANC visits (but not more than two) were more likely to have a healthy weight baby (OR 4.39; 95% CI 1.36–14.15).

**Conclusion:**

The low attendance for ANC, combined with a positive relationship between attendance and perinatal outcomes for the women in the Kwale region highlight the need for further research to understand reasons for attendance and non-attendance and also for strategies to be put in place to improve attendance for ANC.

## Background

"The antenatal period clearly presents opportunities for reaching pregnant women with a number of interventions that may be vital to their health and well-being and that of their infants" [1]. The putative benefits of antenatal care (ANC) to babies include increased growth, reduced risk of infection and increased survival [2]. Some elements of the ANC package (tetanus toxoid, screening for pre-eclampsia, screening and treatment of asymptomatic bacteriuria and syphilis) have been shown to be cost-effective in a Sub-Saharan African context [3]. Although it cannot be claimed that ANC is *the *solution to high maternal and perinatal mortality in the developing world (since "few life-threatening complications can be prevented antenatally" [1]) ensuring the provision of ANC may help progress to the Millennium Development Goals for maternal and child mortality [4].

There have been a number of studies in developing and transitional countries that have shown positive effects of ANC on perinatal outcomes, including reduced rates of pre-term labour [5], low birthweight [5,6] and also perinatal death [6,7]. However much of the research debate has been on the optimal number of ANC visits [8,9], which health-care professionals should provide care [10,11] and the content of the ANC visits [10,12,13]. The WHO now recommends four ANC visits for low risk pregnancies and prescribes the evidence-based content for each visit [14]. As well as the direct effect of ANC on perinatal outcomes (i.e. health benefits arising from the care itself), there may also be an indirect benefit associated with ANC, since women attending ANC are more likely to have their delivery assisted by a professional health care provider [1] or in a health facility [15].

There is evidence that the majority of pregnant women in Kenya have access to ANC, with levels of access (at least one ANC visit) varying from 76% to 92% between different surveys [1,16]. However in 1998, only 65% of women in Kenya reported four or more visits, with most visits occurring in the 2^nd ^(43%) or 3^rd ^(42%) trimesters [1]. A more recent study also highlights a tendency towards 'late' attendance for the first ANC visit in Kenya [15] and coverage in Sub-Saharan Africa as a whole lags behind other developing regions [17]. There are also concerns over the quality of the ANC provided in Sub-Saharan Africa: the figures regarding attendance do not indicate the specific quality or content of the ANC received.

The largest body of evidence regarding the personal characteristics that determine ANC utilisation comes from a DHS survey of 45 developing countries carried out in 2002 [1]. This survey found that educated, wealthy women living in urban areas were more likely to receive ANC than those who lived in rural areas and/or were poor financially. Similar findings with respect to residence and education are reported in the 2003 Kenya Demographic and Health Survey [16]. In their survey in rural Western Kenya, van Eijk et al. [15] report that socio-economic status (SES) and education are also associated with uptake of ANC.

The study reported here is the first stage in the Aga Khan Health Services Kenya/Community Health Department Community-based Health Information System (CBHIS) project. A particular strength of this project lies in the detail of personal information collected. This project is part of a larger, health-facility based programme of strengthening health management information systems (HMIS), which began in 2003. The main objective of the CBHIS project is to assess the extent to which the data collected as part of the larger HMIS programme are a true reflection of what actually happens at community level. In this paper, the community level data were analysed to test two primary hypotheses: ANC is associated with (1) increased likelihood of a live, rather than a stillbirth and (2) increased likelihood of a 'healthy' weight baby (at least 2.5 kg). In addition, this paper explores the determinants of attendance at ANC and the relationship between attendance and behavioural decisions including place of delivery.

## Methods

The current study used a cohort survey. We report on all women with a birth outcome between 1^st ^August 2004 and 31^st ^July 2005.

### Study Area

Kwale is one of the seven districts of the Kenyan Coast province and consists of five administrative divisions, Samburu, Kinango, Matuga, Kubo and Msambweni. The current study is based in all the villages that form the catchment areas for two out of the ten dispensaries in Samburu (Kafunduni and Mazeras) and three out of the eleven in Matuga (Matuga, Magodzoni and Mazumalume). The two divisions were selected on criteria relating to terrain and population density and because Aga Khan Health Services has historically provided support to the two dispensaries in Samburu, as part of a Health Systems Strengthening project between 1997–2000. The three dispensaries in Matuga were matched to those in Samburu on the basis of population size.

Both Matuga and Samburu have a combination of plains and hills, with two rainy seasons (March to June and August to November). The main subsistence crops are maize and cassava, with other fruit trees also grown as cash crops. The history of the people inhabiting the study areas owes allegiance to the Mijikenda ancestry, in which pregnant women are granted leave from their matrimonial home to return to their parental home to deliver. The level of education is fairly low in both divisions, with secondary schools only in Matuga.

### Data collection

Data collection in the study areas was undertaken through a process known as 'continuous registration'. Each household in the participating dispensary catchment areas was visited every four months and interviewed by one of the field enumeration teams. The four-monthly visit schedule was primarily determined by pragmatic reasons, in terms of what could be achieved by the field enumeration teams. Data were collected on household members, marital status, socio-economic status, births, deaths, child growth monitoring, family planning, ANC for pregnant women and migration. ANC cards were checked where available. A copy of the data collection proforma is available on request from SS. The response rate was 99.86% (8 households out of 5,789 refused).

In households where there were pregnant mothers, the enumerators calculated the expected date of delivery (EDD). Calculation of the EDD used the first day of the woman's last menstrual period as a reference and assuming conception occurred 14 days after this date. For deliveries not in a formal health facility, enumerators also returned to the household at the time of the EDD so that birthweight could be recorded within 48 hours of delivery. Birthweight was measured by the enumerators using a digital weighing scale. The enumerators encouraged women to attend for ANC, as it was considered inappropriate not to do so. However, no incentives to attend were offered and this encouragement was not considered an 'intervention' as such.

Data collection was preceded by a series of meetings with local administration officers and village elders to seek their active involvement in the project. These meetings were also used to verify the village boundaries. Twelve field enumerators were recruited for the data collection. The enumerators were required to have at least secondary education (9–10 years schooling), to have come from the dispensary catchment area where they were to work and be able to communicate in the local language. Training for the enumerators consisted of a 5-day course at the Aga Khan hospital in Mombasa. The training was led by a qualified nurse-midwife and included familiarisation with the data collection tools, ANC and child health cards and methods of calculating EDD and weighing babies. The initial training was followed by regular supervision in the field to provide on-site assistance and regular meetings between the enumerators and supervisors.

Data management began with an initial check by the enumerator and a further check by the field assistant. The data were subsequently entered into a database to allow demographic changes occurring between visits to be updated. Data were double entered by two members of the data entry team to check accuracy and were also cleaned at this stage. Data analysis was undertaken using Stata version 7. Descriptive statistics were used to explore differences in background characteristics, behavioural decisions and perinatal outcomes across the five dispensaries. Details of these variables are shown in Table 1. Univariate analyses were used to explore the determinants of attendance at ANC, the association between ANC and other behavioural decisions and the determinants of the two key outcomes: pregnancy outcomes and birthweight. Results are shown as odds ratios (OR) with 95% confidence intervals (CI). Pregnancy outcomes are dichotomised into live birth and stillbirth. A baby is a 'healthy' weight if it is at least 2.5 kg when weighed within 48 hours of delivery.

**Table 1:** Variables included in the analyses

**Background Characteristics**	**Behavioural Decisions**	**Perinatal Outcomes**
Distance from dispensary a) Binary – less or more than 5 km b) Actual distance in km	Antenatal care a) At least one visit b) Number of visits if >0 There were insufficient data to assess the timing of ANC	Pregnancy outcome Live birth vs. stillbirth
SES a) Wealth Quintile (using the World Bank method [21]) b) Income (mean reported income over three visits) There was no correlation between these measures (χ^2 ^= 126, p < 0.001)	Tetanus Typhoid (TT) and Sulfadoxine-Pyrimethamine (SP) doses The number of TT and SP doses are perfectly correlated and hence are considered as a single variable	Healthy Weight Healthy ≥ 2.5 kg
Education	Use of an insecticide-treated mosquito net (ITMN)	
Mother's age	Who assisted delivery	
Gravidity Dummy variable to indicate women who are primigravidae	Where delivery took place A formal health facility is any of the 5 levels of health facility provided in Kenya (Dispensaries/Clinics, Health centres, primary hospitals, secondary hospitals and tertiary hospitals). In 2004, there were 4,767 health facilities in Kenya, 362 of which are in the Coast province [22]. Dispensaries are staffed by enrolled nurses, public health technicians and dressers (medical assistants) [22].	

### Data analysis

Multivariate logistic regression models were then estimated, beginning with a full model that included all possible independent variables (and also dummy variables for the dispensaries). The model with the number of ANC visits as the dependent variable used an ordered logistic regression model. A process of backwards-stepwise elimination was used, with variables with a p-value greater than 0.05 excluded from subsequent model(s). Results are shown as odds ratios (OR) with 95% confidence intervals (CI). Where multicollinearity was suspected, Stata's collinearity test was performed to identify the appropriate variable. Likelihood ratio tests were performed to test whether the reduced models compromised goodness of fit. Two sets of multivariate models for ANC and pregnancy outcome were specified, including distance from the dispensary firstly as a dichotomous variable (using 5 km as the cut point) and secondly as a continuous variable.

## Results

Data are reported for 1,562 perinatal outcomes in the catchment areas of five Ministry of Health dispensaries, identified from visits to 5,781 households. Descriptive statistics across the dispensaries are shown in Table 2. The number of women reporting attending for at least one ANC visit (32%) is much lower than in previous studies of access to ANC in Kenya. 'Good' pregnancy outcomes were reported for 90% of the women, with 6% stillbirths. Of babies born alive, 92% were of a 'healthy' weight. One dispensary with apparently contradictory data is Matuga: women report low incomes, but a relatively high proportion are in the highest wealth quintile and have had at least secondary education. The Matuga dispensary area is close to Kwale town, and there is fairly good access to schools and other facilities there. Hence women from Matuga are more likely to have had access to secondary education and also to assets that are included within the derivation of wealth quintiles, even though the area is generally poor.

**Table 2:** Background, behavioural and outcome variables by dispensary

	**Dispensary Name**					
	**Kafuduni**	**Magodzoni**	**Matuga**	**Mazeras**	**Mazumalume**	**Total**

**Location**	**Samburu**	**Matuga**	**Matgua**	**Samburu**	**Matuga**	

**Number of Observations**	508	159	273	432	190	1562
**Distance from dispensary**						
Distance from dispensary, km (mean, sd) (n = 1,562)	5.15 (3.70)	3.29 (2.22)	2.50 (1.40)	3.68 (5.49)	5.68 (6.04)	4.15 (4.39)
Distance from dispensary, km (median, IQR) (n = 1,562)^1^	4 (3–7)	3 (2–4)	2 (2–3)	4 (1.1–5)	5 (2–8)	4 (2–5.1)
Less than 5 km (%) (n = 1,562)	56.1	81.8	94.5	73.4	48.9	69.3
**Wealth Quintile (%) (n = 1,535)**						
1 (most poor)	22.7	15.7	13.0	22.3	10.0	18.6
2	28.2	15.7	15.2	20.8	10.0	20.4
3	20.7	27.7	13.0	13.1	39.5	20.3
4	15.1	23.3	30.7	17.9	22.6	20.4
5 (least poor)	13.3	17.6	28.2	25.9	17.9	20.3
**Income (Kenyan Shillings/month)**^2^**(%) (n = 1,504)**						
<2,500	26.1	61.6	81.7	12.1	54.6	38.9
2,501–5,000	40.3	20.5	9.2	47.0	32.2	33.8
5,001–7,500	13.4	10.6	5.0	22.8	7.1	13.5
7,501–10,000	10.3	5.3	2.3	14.3	2.2	8.5
>10,000	10.0	2.0	1.9	3.8	3.8	5.3
**Education (%) (n = 1,562)**						
None	42.3	42.2	36.6	39.4	48.4	41.2
Primary	55.5	54.7	56.8	56.5	45.8	54.7
Secondary or above	3.2	3.2	6.6	4.2	5.7	4.1
**Mothers' age (n = 1,562)**						
Mean (sd)	22.7 (7.36)	22.4 (6.55)	22.9 (7.89)	22.1 (6.70)	22.2 (6.57)	22.5 (7.11)
**Gravidity (n = 1,418)**						
Primigravidae (%)	22.3	21.9	16.3	19.6	18.5	20.0
**Behavioural Decisions**						
**ANC Visits (n = 1,562)**						
At least one ANC Visit reported (%)	30.1	34.6	30.8	36.6	29.5	32.4
Number of ANC Visits if >0 (mean, sd)	2.42 (1.06)	2.33 (0.84)	1.85 (0.70)	2.31 (0.96)	2.11 (1.04)	2.25 (0.97)
**Number of TT and SP injections (%)**^3^**(n = 1,554)**						
0	68.4	64.8	69.2	62.7	69.0	66.7
1	8.7	13.2	18.7	13.2	14.4	12.9
2	22.7	22.0	12.1	24.1	16.6	20.4
**Frequency of sleeping under a ITMN (%) (n = 1,562)**						
Never	93.7	94.3	96.3	97.0	96.8	95.5
Sometimes	1.6	0	0.4	1.4	0	1.0
Frequently	4.7	5.7	3.3	1.6	3.2	3.5
**Birth Variables**						
**Person assisting delivery (%) (n = 1,562)**						
Doctor/Nurse	16.5	26.4	19.8	19.0	22.6	19.5
Trained TBA	16.3	23.3	14.7	19.2	16.8	17.6
Untrained TBA	17.5	19.5	11.4	20.1	21.6	17.9
Friend/Relative/Self	40.1	28.3	45.4	37.7	36.3	40.7
Other	3.5	2.5	8.8	3.9	2.6	4.4
**Place of delivery (%) (n = 1,562)**						
Formal health facility	18.1	27.7	21.3	20.1	24.7	21
Home	78.9	68.6	76.9	77.8	71.6	76.3
Other	3	3.8	1.8	2.1	3.7	2.7
**Birth Outcomes**						
**Pregnancy outcome (n = 1,562)**						
Live birth	86.4	95.6	93.0	91.2	86.3	89.8
Still birth	6.9	3.8	5.1	6.0	5.8	5.9
Miscarriage	6.1	0.6	1.8	2.3	6.3	3.8
Abortion	0.6	0	0	0.5	1.6	0.5
**Birth Weight (n = 1,408)**						
Weight if measured within 48 h, kg (mean, sd)	3.01 (0.64)	3.05 (0.51)	3.03 (0.44)	3.10 (0.65)	2.98 (0.54)	3.04 (0.59)
Underweight (% <2.5 kg)	11.6	3.4	2.9	6.4	13.6	7.9

Notes:

1. The median distance is shown since the data are positively skewed.

2. There are approximately 125 Kenyan Shillings to £1.

3. The number of TT and SP doses are perfectly correlated.

### Determinants of attendance for ANC

Table 3 shows the results of the univariate and multivariate analyses for two ANC outcomes: the percentage of women with at least one ANC visit and the number of ANC visits if at least one visit was made. Only one background variable is statistically significant at p < 0.05 for both outcomes: the distance of the woman's home from the dispensary. Two measures of distance are used: whether the woman's home is within 5 km of the dispensary and the actual distance of the dispensary from home. The results for the two outcomes reveal an interesting pattern: while women living closer to the dispensary are more likely to have at least one ANC visit, among those women with at least one visit, the number of ANC visits increases as distance from the dispensary increases. The univariate analysis also shows that women with at least secondary education are more likely to have had at least one ANC visit (unadjusted OR 1.82, 95% CI 1.04–3.07). However the level of education was only retained in one multivariate model for the dichotomous ANC outcome and was not significant when considering the number of ANC visits. The results of the multivariate models suggest that women from Matuga are less likely to have ANC and also report a lower number of visits than women from the reference dispensary area of Kafunduni, while women from Mazumalume report a lower number of visits. None of the multivariate models offer a particularly good fit for the data, indicating that there are other determinants of attending ANC not identified by the survey.

**Table 3:** Determinants of attendance for antenatal care

	**At least 1 ANC Visit (OR, 95% CI) (n = 1,562)**			**Number of ANC Visits if >0 (OR, 95% CI) (n = 506)**		
	Univariate	Multivariate Model 1	Multivariate Model 2	Univariate	Multivariate Model 1	Multivariate Model 2

**Background Variables**						
**Distance from dispensary**						
**(n = 1,562)**						
Less than 5 km	reference	reference		reference	reference	
More than 5 km	0.32 (0.25–0.42)	0.29 (0.22–0.39)		17.63 (9.87–31.19)	18.00 (9.97–32.46)	
Actual Distance	0.71 (0.67–0.75)		0.68 (0.64–0.72)	1.42 (1.31–1.55)		1.46 (1.33–1.60)
**Wealth Quintile (n = 1,535)**						
1 (most poor)	reference			reference		
2	1.45 (1.04–2.02)			1.22 (0.75–1.97)		
3	0.86 (0.61–1.21)			0.90 (0.53–1.52)		
4	0.87 (0.62–1.22)			0.95 (0.57–1.58)		
5 (least poor)	1.12 (0.80–1.57)			0.98 (0.60–1.62)		
**Income (Kenyan**						
**Shillings/month) (n = 1,504)**						
<2,500	reference			reference		
2,501–5,000	0.85 (0.66–1.10)			1.39 (0.94–2.03)		
5,001–7,500	1.29 (0.93–1.78)			1.38 (0.87–2.18)		
7,501–10,000	1.06 (0.71–1.58)			1.08 (0.61–1.90)		
>10,000	0.63 (0.37–1.08)			1.30 (0.51–3.32)		
**Education (n = 1,562)**						
None	reference	reference		reference		
Primary	1.11 (0.89–1.38)	1.15 (0.92–1.44)		1.03 (0.75–1.44)		
Secondary or above	1.82 (1.04–3.07)	1.83 (1.06–3.15)		0.99 (0.51–2.01)		
**Mothers' age (n = 1,562)**						
Age	0.99 (0.98–1.01)			1.00 (0.98–1.02)		
**Gravidity (n = 1,418)**						
Primigravidae	0.91 (0.69–1.21)			0.72 (0.47–1.09)		
**Dispensary**						
Kafuduni	reference	reference	reference	reference	reference	reference
Magodzoni	1.23 (0.84–1.79)	0.93 (0.63–1.37)	0.74 (0.49–1.11)	0.88 (0.51–1.51)	1.30 (0.75–2.26)	1.06 (0.61–1.82)
Matuga	1.03 (0.75–1.42)	0.67 (0.48–0.93)	0.49 (0.35–0.69)	0.38 (0.23–0.60)	0.58 (0.35–0.95)	0.45 (0.28–0.74)
Mazeras	1.34 (1.02–1.76)	1.10 (0.83–1.46)	0.87 (0.64–1.17)	0.86 (0.57–1.30)	1.22 (0.80–1.86)	1.23 (0.84–1.94)
Mazumalume	0.97 (0.67–1.40)	1.05 (0.72–1.54)	1.05 (0.70–1.58)	0.56 (0.31–1.00)	0.54 (0.30–0.97)	0.47 (0.26–0.85)
**Pseudo R**^2^		0.047	0.118		0.095	0.082

Multivariate model 1 uses distance from dispensary as a dichotomous variable, Multivariate model 2 uses distance as a continuous variable.

### The association between ANC and behavioural decisions

**Figure 1:**
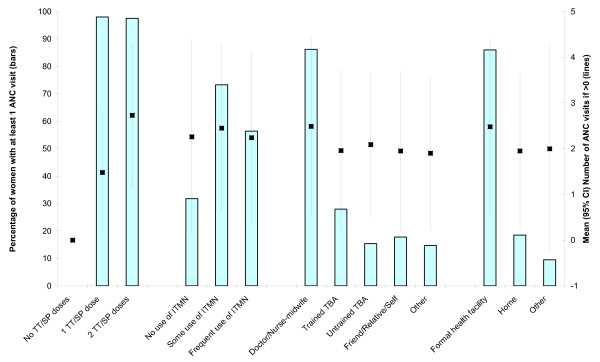
Association between attendance for antenatal care and behavioural decisions.

Figure 1 shows the association between attending ANC and four behavioural decisions: the number of TT and SP doses, use of an ITMN, the person assisting with delivery and place of delivery. The data are also shown in Additional file 1, Table S1. The bars show the percentage of women in each behavioural outcome category reporting at least one ANC visit (left axis scale). Chi squared tests indicate that there is a statistically significant association (p < 0.001) between attending ANC and all four behavioural outcomes (results available from CB). The lines at the centre of each bar indicate the mean and 95% CI number of ANC visits (if at least one was reported) for women in each behavioural outcome category (right axis scale). Women reporting two TT/SP doses rather than one also report more ANC visits (t = 18.1, p < 0.001). One-way ANOVAs for the remaining three behavioural decisions identify statistically significant differences in the number of ANC visits between groups for the person assisting delivery (F = 9.88, p < 0.001) and where delivery occurred (F = 20.51, p < 0.001), but not use of ITMN (F = 0.27, p = 0.766).

### Determinants of pregnancy outcomes (live births)

**Figure 2:**
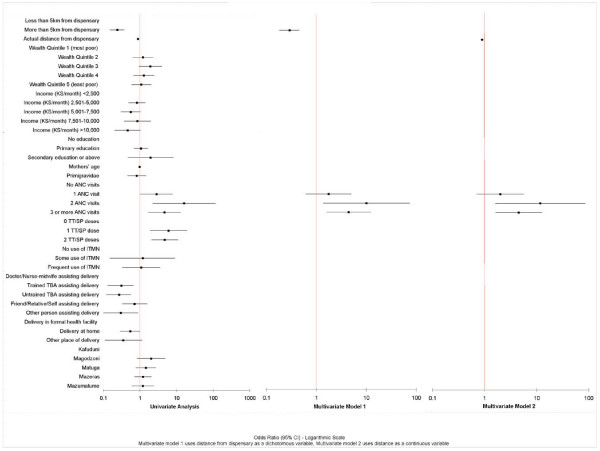
Determinants of live birth (vs. stillbirth).

Figure 2 presents the results of the univariate and multivariate analyses identifying the determinants of live birth (vs. stillbirth), and the data are also shown in Additional file 1, table S2. In the univariate analysis, distance from the dispensary, attendance for ANC, the number of TT/SP doses and the person assisting delivery were all statistically significantly associated (at p < 0.05) with live birth.

The initial multivariate models identified probable multicollinearity between ANC and TT/SP doses and this was confirmed in a collinearity test. Since doses would have been given during ANC visits, the TT/SP variable was dropped from subsequent analyses. The multivariate models suggest that only one ANC visit is not a statistically significant determinant of pregnancy outcome. However women with two or three ANC visits were more likely to have a good outcome, although two visits appears to be more 'effective' than three. The association between outcome and the person assisting delivery and place of delivery were not statistically significant in the initial multivariate models.

### Determinants of birthweight

**Figure 3:**
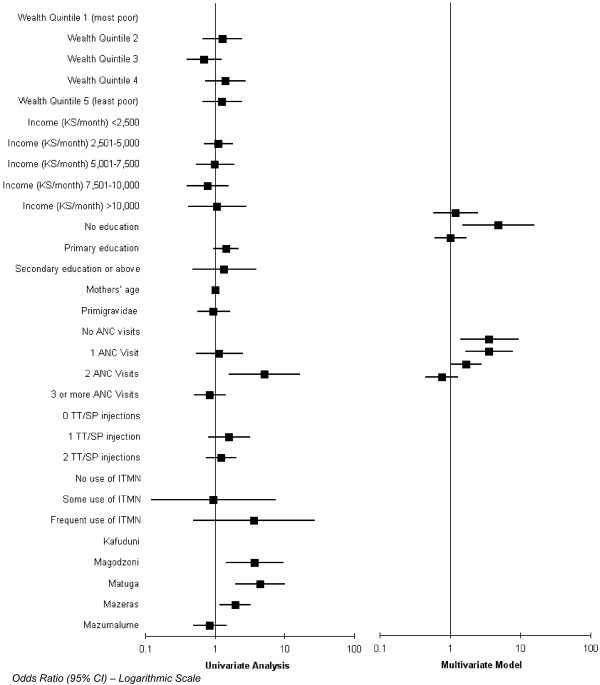
Determinants of 'healthy' weight.

The analysis in this section is based on live births where the baby was weighed within 48 hours (n = 1,340 (96%) of n = 1,403 live births). The results of the univariate and multivariate analyses are shown in Figure 3 and the data are reported in Additional file 1, table S3. Again the TT/SP doses variable was dropped from the multivariate analysis due to multicollinearity. The only statistically significant determinant of whether a baby is a 'healthy' weight is having two ANC visits. Women with three ANC visits were not statistically significantly more likely to have a healthy weight baby. Women in Magodzoni, Matuga and Mazeras were all statistically significantly more likely to have a healthy weight baby compared to women in Kafunduni.

## Discussion

The analyses in this paper have confirmed the association between ANC and perinatal outcomes (pregnancy outcomes and birthweight) in the Kwale region of Kenya, although the percentage of women accessing ANC (32%) is considerably below that found in other surveys [1,16]. This low utilisation of ANC may be expected given that Kwale is a particularly deprived rural area of Kenya. The informal encouragement by enumerators was insufficient to convince many women to attend, although this encouragement was not tested as an intervention to improve attendance. However our data reveal an interesting pattern, whereby two ANC visits is associated with a better outcome than three or more visits. This may be related to general low attendance and hence circumstances where women tend to attend more frequently if they are having problems with their pregnancy.

It is possible that the relationship between ANC and birthweight is affected by the length of pregnancy, since mothers of babies born prematurely would have had less time in which to attend for ANC. However we do not have sufficient data on gestational age in order to test this hypothesis and ascertain the extent of any bias arising from prematurity.

One of the reasons ANC may have a positive association with perinatal outcomes is the association between attending ANC and behavioural decisions, including TT and SP doses, use of an ITMN and decisions regarding place of delivery and who assists the delivery. The associations between ANC and TT/SP doses and use of an ITMN are not surprising as these interventions are part of the ANC care package, and it is unlikely that women would have access to these interventions outside of ANC services. ANC clients are also routinely given iron, folic acid and vitamin supplements, which may impact perinatal outcomes. As noted in previous research [1,15], women who have had ANC are more likely to deliver in a formal health facility and/or in the presence of a doctor or nurse-midwife. Given the low level of attendance for ANC, it is not surprising that the proportion of women delivering in a formal health facility and/or in the presence of a doctor or nurse-midwife is roughly half the 40% found in the 2003 Demographic and Health Survey in Kenya [16]. However deciphering the links between ANC, behavioural decisions and outcomes is always difficult in non-experimental studies. For example, it is possible that differences in the incidence of malaria between dispensaries affected the proportion of low birthweight babies across the dispensaries, but data on the incidence of malaria are inadequate for us to test this hypothesis.

Given the low attendance at ANC by women in the study areas, there is a need for qualitative research to investigate why women do or do not attend for ANC and could help to identify the effect of ANC on behavioural decisions. Qualitative research in a rural area of Zambia, for example, revealed that long distances, lack of transport, user fees, lack of health education and poor quality care deterred women from delivering in a clinic [18]. Three potential factors deserve particular attention. First, there appears to be a significant proportion of women who live some way from the dispensary and who have no ANC. Hence lack of good roads or transport may be the barrier to attendance and this factor should be explored in qualitative research. If this is the case, women in distant areas could be encouraged to attend for ANC by providing transport or arranging an out-reach ANC service in certain areas. Alternatively, an attempt could be made to provide more health facilities to reduce the need for women to travel long distances while pregnant or to consider the use of maternity waiting homes [19].

Second, variations in the content and quality of care received across dispensaries should be investigated. Such variations may help to explain differences in the number of ANC visits between dispensaries: women receiving poor care would be less likely to return for further visits. For example, there is some evidence that women in Samburu are more likely to attend ANC than women in Matuga and this may reflect the previous work of Aga Khan in strengthening health systems in Samburu. The quality of care should be assessed both within the dispensary and also through a survey of women who have received care: it may be the case that women in one area are less satisfied with a given level of care than women in another area, due to differences in expectations [20].

Third, the analyses identified a positive association between secondary education and attendance for ANC (although the association was only statistically significant in one of the multivariate analyses). Campbell et al. [2] suggest that the provision of advice on seeking ANC is an important component of the care package for reducing maternal mortality and this advice can be delivered by a variety of methods. Efforts to encourage women to attend ANC could therefore be targeted at less educated women and could include formal (e.g. employment or school based) or informal education sessions for younger women which include information on ANC and childbirth [2]. Any programs used to encourage use of ANC should be evaluated to identify their effectiveness (or otherwise).

In contrast to previous studies in Kenya [1,15], the analyses reported here did not identify any association between income or SES and attending ANC or perinatal outcomes. Even though two different measures of SES were used in the analyses, it may be the case that another financial variable is affecting decisions of whether to attend ANC and again qualitative research may help to uncover this variable.

The data in this study are limited to two areas within the Kwale region of Kenya, and hence the results may not be generalisable to other regions. The response rate was very high (almost 100%), indicating high precision in the data. However, the data collection process was unable to identify the timing of women's ANC since few knew their dates of conception or ANC visits and therefore it has not been possible to compare ANC timing with previous research in Kenya, or to identify the effect of timing on perinatal outcomes. There were a few sets of twins in the sample (the exact number is not known), and the birthweights of each twin were recorded separately. This may have created a small bias in the data, particularly in terms of birthweight. Furthermore, the explanatory power of the multivariate models was low, indicating that there are a number of other factors affecting ANC attendance and perinatal outcomes. Qualitative research may help to identify these factors and hence identify possible interventions to improve ANC attendance and perinatal outcomes.

## Conclusion

The results reported here highlight the relationship between ANC and good perinatal outcomes in the Kwale region of Kenya, although it is not clear whether this is a causal relationship as attending ANC may be a marker for women who take good care of themselves generally. The results identify several avenues for further research and action to increase the number of women receiving ANC. Plans will now be drawn up by Aga Khan Health Services for interventions to increase access to ANC for women in the Kwale region, which can be evaluated using the current data as a baseline. Such interventions may include preparing community health workers to promote ANC, improving the quality of ANC offered by nurses at the dispensary level and strengthening health systems to ensure the availability of medical supplies. The data collected as part of this study will also be used to health district health teams to plan new facilities or establish mobile clinics to help those living far from the dispensaries to access care.

## Abbreviations

ANC Antenatal Care

CBHIS Community-based Health Information System

CI Confidence Interval

EDD Expected date of delivery

HMIS Health Management Information System

ITMN Insecticide-treated Mosquito Net

OR Odds Ratio

SES Socio-economic Status

SP Sulfadoxine-Pyrimethamine

TBA Traditional Birth Attendant

TT Tetanus Typhoid

## Competing interests

The author(s) declare that they have no competing interests.

## Authors' contributions

At the time the study was conducted, SS directed the Community Health Department and initiated the study. SS and MW planned the data collection process. The data were managed by MW. CB/KK and RL planned the data analysis. CB undertook the data analysis with assistance from KK. CB drafted the paper with assistance from the other authors. All authors approved the final manuscript.

## Pre-publication history

The pre-publication history for this paper can be accessed here:



## Supplementary Material

Additional file 1 Tables of results for the association between attendance for ANC and behavioural decisions (Table 1), determinants of live birth (vs. stillbirth (Table 2) and determinants of 'healthy' weight (Table 3). Click here for file
