# Supplementary material for: Antenatal care and perinatal outcomes in Kwale district, Kenya
Source: BMC Pregnancy Childbirth. 2008 Jan 10;8:2. doi: 10.1186/1471-2393-8-2 (PMC2254374; doi:10.1186/1471-2393-8-2)
Supplement: Additional file 1 — Tables of results for the association between attendance for ANC and behavioural decisions (Table 1), determinants of live birth (vs. stillbirth (Table 2) and determinants of 'healthy' weight (Table 3). [file 1471-2393-8-2-S1.doc]

Additional data table 2: Determinants of live birth (vs. still birth)

|  | **Live birth (OR, 95% CI) (n=1,495)** | | |
| --- | --- | --- | --- |
|  | Univariate | **Multivariate Model 1** | **Multivariate Model 2** |
| **Background Variables** | | | |
| **Distance from dispensary (n=1,495)** | | | |
| Less than 5km | Reference | Reference |  |
| More than 5km | 0.24 (0.15-0.37) | 0.29 (0.18-0.45) |  |
| Actual distance | 0.88 (0.83-0.93) |  | 0.90 (0.85-0.95) |
| **Wealth Quintile (n=1,495)** | | | |
| 1 (most poor) | Reference |  |  |
| 2 | 1.21 (0.64-2.28) |  |  |
| 3 | 1.92 (0.93-3.96) |  |  |
| 4 | 1.28 (0.67-2.43) |  |  |
| 5 (least poor) | 1.09 (0.59-2.03) |  |  |
| **Income (Kenyan Shillings/month) (n=1,495)** | | | |
| <2,500 | Reference |  |  |
| 2,501-5,000 | 0.83 (0.49-1.40) |  |  |
| 5,001-7,500 | 0.56 (0.30-1.03) |  |  |
| 7,501-10,000 | 0.85 (0.37-1.98) |  |  |
| >10,000 | 0.46 (0.20-1.03) |  |  |
| **Education (n=1,495)** | | | |
| None | Reference |  |  |
| Primary | 1.06 (0.69-1.64) |  |  |
| Secondary or above | 1.94 (0.46-8.23) |  |  |
| **Mothers’ age (n=1,495)** | | | |
| Age | 0.98 (0.96-1.01) |  |  |
| **Gravidity (n=1,355)** | | | |
| Primigravidae | 0.81 (0.45-1.46) |  |  |
| **Behavioural Variables** | | | |
| ANC Visits (n=1,495) | | | |
| None | Reference | Reference | Reference |
| 1 Visit | 2.83 (1.02-7.85) | 1.75 (0.61-4.97) | 2.00 (0.71-5.67) |
| 2 Visits | 16.10 (2.23-116.43) | 10.04 (1.37-73.42) | 11.72 (1.61-85.48) |
| 3 or more Visits | 4.67 (1.69-12.88) | 4.43 (1.60-12.29) | 4.54 (1.63-12.67) |
| **Number of TT and SP injections (n=1,495)** | | | |
| 0 | Reference | Dropped | Dropped |
| 1 | 6.03 (1.88-19.26) |  |  |
| 2 | 4.72 (2.04-10.92) |  |  |
| **Frequency of sleeping under a ITMN (n=1,495)** | | | |
| Never | Reference |  |  |
| Sometimes | 1.17 (0.15-9.06) |  |  |
| Frequently | 1.09 (0.33-3.58) |  |  |
| **Birth Variables** | | | |
| **Person assisting delivery (n=1,495)** | | | |
| Doctor/Nurse | Reference |  |  |
| Trained TBA | 0.31 (0.13-0.67) |  |  |
| Untrained TBA | 0.27 (0.12-0.57) |  |  |
| Friend/Relative/Self | 0.71 (0.33-1.55) |  |  |
| Other | 0.30 (0.10-0.89) |  |  |
| **Place of delivery (n=1,495)** | | | |
| Formal health facility | Reference |  |  |
| Home | 0.54 (0.29-1.00) |  |  |
| Other | 0.35 (0.11-1.14) |  |  |
| **Dispensary (n=1,495)** | | | |
| Kafuduni | Reference |  |  |
| Magodzoni | 2.02 (0.83-4.90) |  |  |
| Matuga | 1.45 (0.76-2.74) |  |  |
| Mazeras | 1.21 (0.71-2.04) |  |  |
| Mazumalume | 1.19 (0.59-2.40) |  |  |
| Pseudo R^2^ |  | 0.0939 | 0.0724 |

### Additional data table 3: Determinants of ‘healthy’ weight

|  | **‘Healthy’ Weight >2.5kg (OR, 95% CI) (n=1,340)** | |
| --- | --- | --- |
|  | Univariate | **Multivariate Model** |
| **Background Variables** | | |
| **Wealth Quintile (n=1,340)** | | |
| 1 (most poor) | Reference |  |
| 2 | 1.27 (0.66-2.42) |  |
| 3 | 0.69 (0.39-1.23) |  |
| 4 | 1.41 (0.73-2.72) |  |
| 5 (least poor) | 1.26 (0.66-2.41) |  |
| **Income (Kenyan Shillings/month) (n=1,340)** | | |
| <2,500 | Reference |  |
| 2,501-5,000 | 1.11 (0.69-1.79) |  |
| 5,001-7,500 | 0.99 (0.53-1.85) |  |
| 7,501-10,000 | 0.77 (0.39-1.55) |  |
| >10,000 | 1.06 (0.41-2.78) |  |
| **Education (n=1,340)** | | |
| None | Reference |  |
| Primary | 1.42 (0.95-2.13) |  |
| Secondary or above | 1.35 (0.47-3.89) |  |
| **Mothers’ age (n=1340)** | | |
| Age | 1.00 (0.98-1.03) |  |
| **Gravidity (n=1,215)** | | |
| Primigravidae | 0.95 (0.56-1.63) |  |
| **Behavioural Variables** | | |
| ANC Visits (n=1,340) | | |
| None | Reference | Reference |
| 1 Visit | 1.14 (0.53-2.44) | 1.18 (0.55-2.53) |
| 2 Visits | 5.12 (1.59-16.43) | 4.39 (1.36-14.15) |
| 3 or more Visits | 0.84 (0.50-1.41) | 0.91 (0.54-1.54) |
| **Number of TT and SP injections (n=1,340)** | | |
| 0 | Reference | Dropped |
| 1 | 1.57 (0.79-3.10) |  |
| 2 | 1.23 (0.75-2.04) |  |
| **Frequency of sleeping under a ITMN (n=1,340)** | | |
| Never | Reference |  |
| Sometimes | 0.95 (0.12-7.35) |  |
| Frequently | 3.61 (0.49-26.51) |  |
| **Dispensary (n=1,340)** |  |  |
| Kafuduni | Reference | Reference |
| Magodzoni | 3.71 (1.45-9.51) | 3.54 (1.38-9.08) |
| Matuga | 4.42 (1.97-9.93) | 4.04 (1.79-9.10) |
| Mazeras | 1.94 (1.16-3.22) | 1.91 (1.14-3.18) |
| Mazumalume | 0.83 (0.48-1.44) | 0.85 (0.49-1.48) |
| Pseudo R^2^ |  | 0.0543 |
